# Research on an SICM Scanning Image Resolution Enhancement Algorithm

**DOI:** 10.3390/s24113291

**Published:** 2024-05-22

**Authors:** Zhenhua Quan, Shilin Xu, Xiaobo Liao, Bin Wu, Liang Luo

**Affiliations:** School of Information Engineering, Southwest University of Science and Technology, Mianyang 621010, China; xushilin@swust.edu.cn (S.X.); liaoxiaobo@swust.edu.cn (X.L.); wubin@swust.edu.cn (B.W.); luoliang@swust.edu.cn (L.L.)

**Keywords:** scanning ion conductance microscopy, scanning imaging resolution enhancement, NEDI

## Abstract

Scanning ion conductance microscopy (SICM) enables the non-invasive three-dimensional imaging of live cells and other structures in physiological environments. However, when imaging complex samples, SICM faces challenges such as having a low temporal resolution during slow scanning and a reduced signal-to-noise ratio during fast scanning, making it difficult to simultaneously improve both temporal and spatial resolution. To address these issues, this paper proposes an algorithm for enhancing image resolution under high-speed scanning. Firstly, scanning images are preprocessed using a median filtering algorithm to remove the salt-and-pepper noise generated during high-speed scanning. Next, the Canny edge detection algorithm is employed to extract the edges of the image targets. To avoid blurring the edges, the new edge-directed interpolation (NEDI) algorithm is then used to fill the edges, while non-edge areas are filled using bilinear interpolation, thereby enhancing the image resolution. Finally, the peak signal-to-noise ratio (PSNR) and structural similarity index (SSIM) are used to analyze the imaging of articular chondrocytes. The results show that under a scanning speed of 480 nm/ms, the proposed algorithm improves the temporal resolution of imaging by 60% compared to traditional 2× resolution imaging, increases the peak signal-to-noise ratio of the scanning images by 7 dB, and achieves a structural similarity of 0.97. Therefore, the proposed algorithm effectively removes noise during high-speed scanning and improves the SICM scanning imaging resolution, thereby avoiding the reduction in temporal resolution when scanning larger resolution samples and effectively enhancing the performance of SICM scanning imaging.

## 1. Introduction

Micro-nano-imaging technology has been a hot topic in scientific research, especially in the detection and characterization of micro-nano-structures, where the selection of appropriate detection techniques and instrumentation is crucial [1]. With the advancement of research in life sciences and other fields, traditional optical and electron microscopes are no longer sufficient to meet the observation requirements under certain circumstances. Therefore, scanning ion conductance microscopy (SICM), as an emerging Scanning Probe Microscopy (SPM) technique, was first proposed by Hansma et al. in 1989 [1]. This technology enables the three-dimensional imaging of conductors, semiconductors, and non-conductors, with its greatest advantage lying in its ability to perform non-contact, stress-free scanning imaging of biological samples, facilitating real-time dynamic studies under physiological conditions [2].

Research on the performance of SICM mainly focuses on two key aspects: optimizing the SICM scanning modes and improving scanning speed. However, SICM commonly suffers from a slow imaging speed and a long acquisition time, failing to meet real-time requirements. To address this issue, scholars have conducted extensive research. In 2014, the team led by Korchev at Imperial College London [3] invented a dual piezoelectric ceramic probe structure to accelerate the probe’s lifting speed and thus increase its scanning rate. However, this improvement increased the complexity and cost of the probe. In 2017, the team led by Zhuang Jian at Xi’an Jiaotong University [4] proposed a pre-scanning mode, involving horizontal movement when the probe approaches the measurement point, predicting surface topography changes by monitoring ion current variations, thereby reducing the probe retraction height, and shortening the scanning path. In 2019, the Schaffer team [5] adopted millimeter-level piezoelectric ceramics to expand the measurement range to 25 × 25 mm^2^, improving the scanning speed but increasing equipment costs to some extent. However, with the increase in the probe scanning speed, noise interference with the ion current during the scanning process increases, resulting in more noise in the scanned images and decreased imaging quality [6]. In 2017, Wang Xiaodong [7] combined morphology image spectra and Sobel edge detection to construct a frequency-positioning band-stop filter to remove periodic noise in SPM. However, post-processing led to blurred image edges. In 2022, Chen Yan et al. [8] proposed a SICM image denoising algorithm combining wavelet transform and bilateral filtering. However, after denoising, the edge preservation of the image was incomplete, and the image resolution [9] did not improve.

Currently, although SICM measurement methods have made significant progress in terms of their being systematic and their applicability, the imaging process of SICM involves scanning each pixel one by one, resulting in a slow imaging speed and low imaging efficiency when dealing with complex sample morphologies. Low-speed scanning images have slow imaging speeds and low imaging efficiency. However, high-speed scanning leads to a decrease in the signal-to-noise ratio. It is challenging for SICM to balance between improving time and spatial resolution. SICM compressed sensing undersampling imaging is an emerging and unique imaging method. This method reduces the sampling rate of images and utilizes the sparsity or low rankness of signals to effectively reconstruct the original signal from limited sampling data. The significant advantage of the compressed sensing undersampling imaging method is that it significantly reduces sampling data, reduces data transmission and storage requirements, and, to some extent, maintains an accurate reconstruction of the original signal, thereby shortening the SICM scanning time and improving imaging efficiency. However, reconstructed images often contain a certain amount of noise and have lower resolution.

This paper proposes an algorithm specifically for denoising and enhancing the resolution of SICM high-speed undersampling reconstruction images. First, preprocess the reconstructed image to remove noise using median filtering to ensure that the subsequent edge detection is not affected by “pseudo-edges”. Then, extract the edge information of the denoised image using edge detection algorithms and expand the pixels of the denoised image. Finally, use Noise-Enhanced Data Interpolation (NEDI) technology to interpolate the image (NEDI for edges and bilinear interpolation for non-edges), ultimately achieving denoising and the resolution enhancement of the image. This algorithm effectively overcomes the problems of image noise and resolution reduction that may be introduced by compressed sensing undersampling methods, providing more reliable and clear images while meeting real-time requirements.

## 2. SICM Imaging Temporal Resolution and Noise Analysis

### 2.1. Principles of SICM Imaging

**Figure 1:**
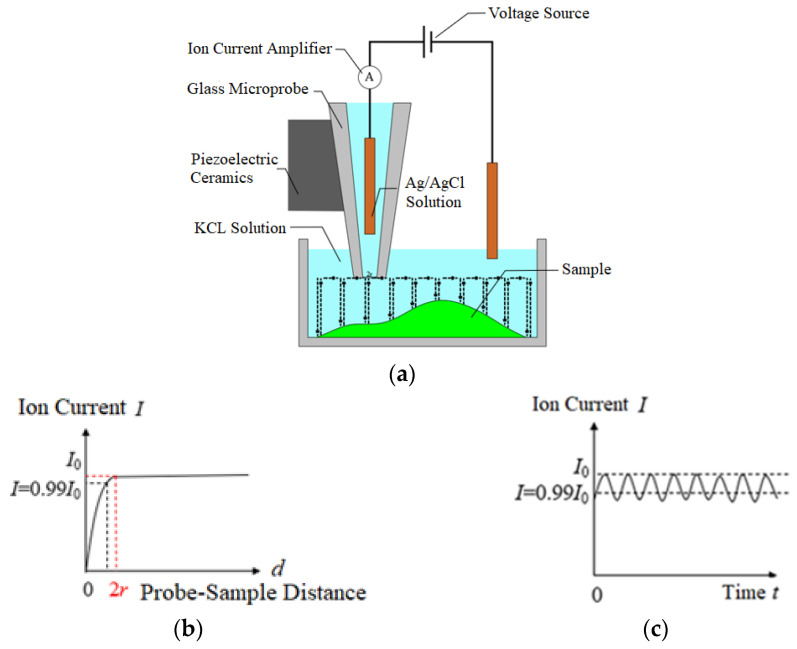
The SICM jump mode’s imaging principle. (**a**) SICM Measurement Configuration. (**b**) Approach Curve. (**c**) Ion Current Change Curve.

SICM is a technology that uses a scanning probe for in situ imaging [10]. Currently, according to the classification based on the scanning probe’s motion trajectory [11], SICM scanning modes can be divided into continuous mode (constant mode) and hopping mode [12]. Due to its suitability for scanning entire cells and arbitrary shapes, the hopping mode is the most widely used. Figure 1 illustrates the imaging principle in the hopping mode. The imaging setup in the hopping mode is shown in Figure 1a, where the sample is immersed in a KCl solution, a glass microprobe filled with the same solution is used for topographical scanning, and two Ag/AgCl electrodes are inserted into the solution and the glass microprobe. Under the action of a voltage source, the two electrodes cause the directional movement of ions in the solution, forming an ion current. Initially, the ion current when the probe is far from the sample is recorded. During the scanning process, the probe gradually approaches the sample surface. When the distance between the probe and the sample surface is approximately equal to the probe tip diameter, the ion current gradually decreases. When the reduction reaches a set threshold (usually 1%), the height of that point is recorded as the sample height. The relationship between the ion current and the probe–sample distance during the decrease in ion current is referred to as the “approach curve,” as shown in Figure 1b. After detecting the imaging point information, the probe retracts to a preset height, moves forward to the next imaging point, and then repeats the downward detection to complete the scan. Throughout the scanning process, the ion current fluctuates within a very small range, approximating a straight line, known as the “ion current change curve” [13], as shown in Figure 1c.

### 2.2. Time Resolution and Noise Analysis in an SICM Voltage Source

In the hopping mode of SICM scanning, the relationship between the ion current and the probe–sample distance during the downward probing process can be determined based on the theoretical model analysis [14]. Equation (1) illustrates this relationship.

(1) $$I(d)=\frac{I_{ref}}{1+\left[3/2\ln\left(r_{o}/r\right)\cdot \tan\theta \cdot r\right]/d}$$

In the equation, where $I_{ref}$ represents the ion current flowing through the probe tip when the probe is away from the sample (reference current), $r_{o}$ is the outer radius of the probe tip, r is the inner radius of the tip, and d is the distance between the probe and the sample. Once the probe is fabricated, and the solution is prepared, values for $I_{ref}$, $r_{o}$, r, and tanθ are constant. Therefore, the ion current I(d) is solely dependent on the distance d between the probe and the sample.

**Figure 2:**
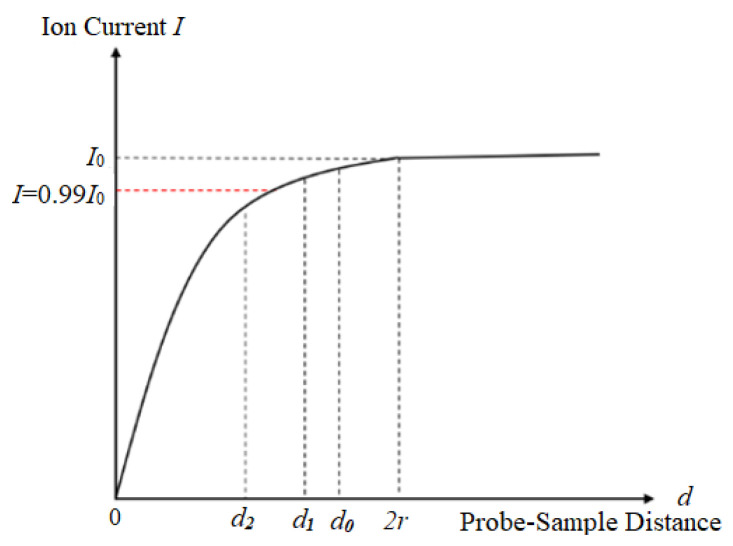
The relationship between the ion current and the probe–sample distance.

In Equation (1), the magnitude of the ion current is not only related to the distribution of the solution concentration, the probe’s aperture radius, cone angle, and the distance between the probe tip and the sample, but also closely related to the probe’s working environment. Since the ion current is on the order of picoamperes, it is susceptible to electromagnetic interference in the air, which can lead to noise [15]. At the same time, to improve the temporal resolution of the scan, it is not only necessary for the probe’s jump height to be as small as possible, but also for its movement speed to be as fast as possible [16]. Figure 2 shows the relationship curve between the SICM’s ion current and the probe–sample distance.

During the probe movement process, the frequency of ion current collection remains constant. Assuming the motion speed within the collection period is v, then the distance covered during the collection period is represented by Equation (2).

(2) D=T×v

According to Equation (2), it is evident that as the speed v increases, the distance D covered during motion becomes larger, resulting in a smaller distance between the probe tip and the sample. If the speed is too high, it may cause the probe tip to meet the sample, potentially damaging it. Referring to Figure 2, assuming the initial position of the probe is $d_{0}$ and the ion current’s change rate is $I_{k}$, when the speed is $v_{slow}$, the probe moves to position $d_{1}$ within one sampling period. Similarly, when the speed is $v_{fast}$, the probe moves to position $d_{2}$ within one sampling period. Therefore, during the approach to the sample at a speed of $v_{slow}$, the ion current’s change rate is given by Equation (3).

(3) $$I_{k1}=\frac{\Delta I_{1}}{\Delta d_{1}}=\frac{I_{d0}-I_{d1}}{d_{0}-d_{1}}$$

The ion current’s change rate during the approach to the sample at speed $v_{fast}$ is as given by Equation (4).

(4) $$I_{k2}=\frac{\Delta I_{2}}{\Delta d_{2}}=\frac{I_{d0}-I_{d2}}{d_{0}-d_{2}}$$

By comparing Equations (3) and (4), it can be inferred that $I_{k2}>I_{k1}$, meaning that within the same scanning time, a faster scanning speed results in a faster change in the ion current. If the decrease in the ion current exceeds the set detection threshold ($I_{d}<0.99I_{0}$), it will lead to the formation of scanning noise points [17]. Therefore, a faster scanning speed also increases the probability of noise appearing in the scanning image.

## 3. Image Processing

### 3.1. Processing Workflow

**Figure 3:**
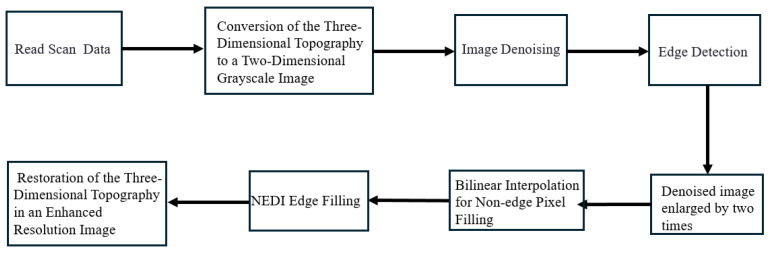
Image processing workflow.

This paper analyzes the characteristics of reconstructed images obtained through high-speed undersampling scanning and proposes a method to enhance image resolution. The specific processing workflow is illustrated in Figure 3.

First, information is extracted from the scanning data, and the three-dimensional morphology of the reconstructed image is converted into a two-dimensional grayscale image. Next, the grayscale image containing noise is subjected to denoising processing, and the contour edges of the image are extracted using edge detection algorithms. Subsequently, the denoised image is subjected to pixel expansion processing, distinguishing between edges, filling missing non-edge pixels using bilinear interpolation, and employing NEDI technology to fill edge pixels, thereby obtaining a two-dimensional image with enhanced resolution and reduced noise. Finally, the expanded image is transformed into three dimensions to obtain the enhanced resolution of the three-dimensional morphology.

### 3.2. Image Denoising

**Figure 4:**
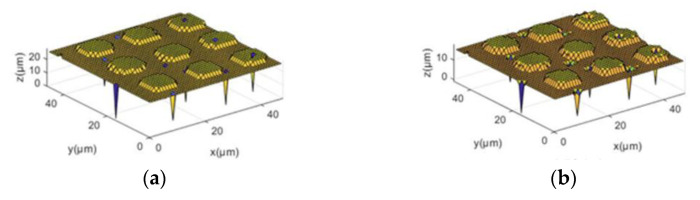

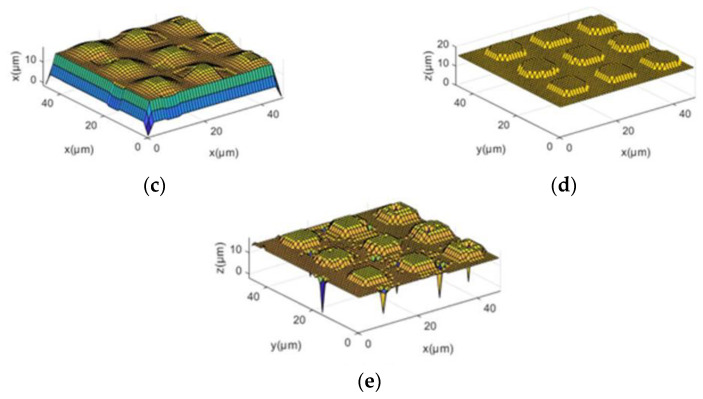
A comparison of the denoising effects of typical filtering algorithms. (**a**) Reconstructed morphology. (**b**) Gaussian Filtering (Kernel Size 3 × 3). (**c**) Mean Filtering (Kernel Size 3 × 3). (**d**) Median Filtering (Kernel Size 3 × 3). (**e**) Wavelet Filtering (Wavelet Type haar).

In general, image denoising methods can be categorized into three main types, including filter-based methods, model-based methods, and learning-based methods [18]. Filter-based methods are commonly employed for local structure processing, effectively preserving details within the image, particularly excelling in the removal of local noise. Additionally, these methods typically feature adjustable parameters, allowing flexible tuning to different noise characteristics and image types, thereby enhancing adaptability. Considering the noise characteristics of scanning ion conductance microscopy (SICM) images, this paper conducts a comparative analysis of various filter-based denoising algorithms. Figure 4 illustrates the results of several typical denoising algorithms applied to scanned images. Table 1 provides the peak signal-to-noise ratios for images processed using each algorithm.

Figure 4a shows the three-dimensional morphology of the sample, from which it can be observed that the surface is relatively smooth overall, but there are still some sharp noises, causing the morphology at the noise locations to be obscured. Figure 4b displays the morphology after Gaussian filtering, where the noise is slightly reduced after denoising, but new depressions appear at the contour edges of the sample. Figure 4c presents the morphology after mean filtering, where, although the noise disappears completely after denoising, the edges of the sample morphology exhibit a “slope” shape, indicating that the denoising algorithm has caused damage to the morphology. Figure 4d illustrates the result after median filtering, where the original noise is completely removed, and the morphology remains intact. Figure 4e depicts the morphology after wavelet filtering, and a comparison before and after processing reveals only a slight reduction in noise, indicating poor denoising effects, while new depressions appear on the surface of the morphology.

To objectively compare the effects of each filtering algorithm and select the appropriate one, this paper calculated the peak signal-to-noise ratio of the images under each algorithm, as shown in Table 1. The peak signal-to-noise ratio (PSNR) is a commonly used metric for assessing image quality. It evaluates the denoising effect by measuring the differences between the original image and the denoised image. The calculation formula for the PSNR is given by Equation (10).

From Table 1, it can be observed that the peak signal-to-noise ratio of the original sample morphology is 29.3176, while after Gaussian filtering, mean filtering, median filtering, and wavelet filtering, it becomes 30.5351, 33.4893, 37.1769, and 31.8092, respectively. The calculation results indicate that the peak signal-to-noise ratio increased by approximately 1.2 dB and 2.5 dB after Gaussian filtering and wavelet filtering, respectively, suggesting a weak denoising effect. The peak signal-to-noise ratio increased by 4.1 dB after mean filtering, indicating a relatively good denoising effect. However, after median filtering, the peak signal-to-noise ratio improved by 7 dB, demonstrating the best denoising effect. Based on this analysis, this paper adopts the median filtering denoising algorithm for the scanning images.

After denoising the reconstructed image, the image quality is significantly improved. However, the image resolution remains relatively low, with poor edge discrimination, which affects morphology analysis. Therefore, it is necessary to enhance the image resolution, and the method used is interpolation enlargement. However, due to the large grayscale gradient at the image edges, traditional interpolation may cause edge blurring. Therefore, different algorithms need to be applied to the edges and non-edges to accurately extract the image target edges.

### 3.3. Edge Detection

Edge detection is a fundamental aspect in the field of image processing, playing a crucial role in image segmentation, object recognition, and shape extraction. It is also an important attribute for extracting image features in image recognition [19]. Currently, commonly used image edge detection algorithms include those based on the Sobel operator [20], the Roberts operator [21], the Prewitt operator [22], the Canny operator [23], and the Laplacian of Gaussian (LoG) operator [24]. These algorithms utilize the property of the maximum grayscale gradient at the edges to determine the edges [25].

**Figure 5:**
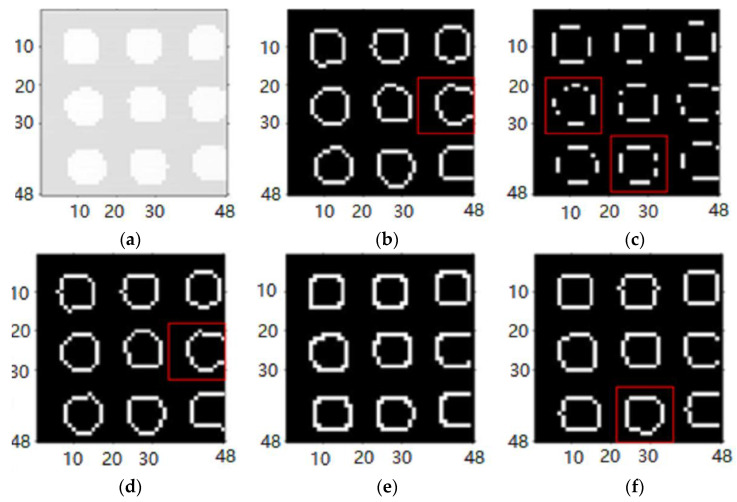
A comparison of the edge extraction effects of common edge detection operators of the ICM jumping mode’s imaging principle. (**a**) Denoised image. (**b**) Sobel Edge Detection. (**c**) Roberts Edge Detection. (**d**) Prewitt Edge Detection. (**e**) Canny Edge Detection. (**f**) LoG Edge Detection.

In this paper, the edge detection operators are individually applied to the denoised images for edge detection, and the detection results are shown in Figure 5.

Figure 5a represents the original denoised image with continuous edge contours. Defects identified in the detection by various algorithms are indicated by red-boxed regions in the image. In Figure 5c, the Roberts operator detects edge contours with incomplete and discontinuous pixels, failing to form a closed contour, resulting in the poorest detection performance. Although Figure 5d, using the Prewitt operator, approximately captures the overall contour, it suffers from a significant number of missing edge pixels, indicating subpar detection performance. While the edge detection results of the Sobel operator in Figure 5b and the LoG (Laplacian of Gaussian) operator in Figure 5f are superior to those of the Prewitt operator, a small number of edge pixels are still missing, and the detection performance falls short of the desired requirements. Notably, Figure 5e, employing the Canny operator, not only successfully detects complete edge contours but also exhibits no missing pixels, surpassing the performance of other operators. Based on this analysis, the Canny operator is chosen in this study as the edge detection algorithm for the denoised images. Following edge extraction using the Canny operator, this paper further employs interpolation to fill in missing pixels, achieving resolution enhancement.

## 4. NEDI Image Resolution Enhancement

The NEDI algorithm, which stands for new edge-directed interpolation [26], considers the relationship between the interpolation coefficients of high- and low-resolution images of the same shape. As a result, the processed image appears to be smooth, and, more importantly, the traditional interpolation-induced blurring of edges is avoided. Therefore, NEDI is used to fill in the missing pixels in the enlarged image.

Due to the matrix inversion involved in the NEDI algorithm, it requires a large amount of computation, leading to longer processing times for image interpolation. Considering this factor, this paper adopts a method where NEDI is applied to the edges and traditional bilinear interpolation is used for non-edges. This approach significantly reduces the computational load and processing time while ensuring edge sharpness, ultimately achieving the goal of enhancing the resolution of the scanning image.

The NEDI calculation formula is given by Equation (5):

(5) $$I(2i+1,2j+1)=\sum_{a=0}^{1}\sum_{b=0}^{1}\alpha_{2a+b}I(2(i+a),2(j+b))$$

where I represents the low-resolution image, i represents the horizontal coordinate of the low-resolution image, j represents the vertical coordinate of the low-resolution image, a represents the X-direction offset of the four neighboring pixels relative to the center point of the pixel to be interpolated, b represents the Y-direction offset of the four neighboring pixels relative to the center point, and $\alpha =R^{-1}r$ represents the interpolation coefficient of the high-resolution image at point $\alpha =R^{-1}r$. After using the Canny operator to extract the edges, this paper further employs interpolation to fill in the missing pixels, achieving resolution enhancement.

(6) $$\alpha =R^{-1}r$$

In the equation, R represents the vector composed of four diagonally adjacent pixels at point $\left(i,j\right)$ in the low-resolution image. r, on the other hand, represents the vector formed by four diagonally adjacent pixels at the interpolation point in the high-resolution image.

The calculation formula for the low-resolution image R is

(7) $$R=\frac{1}{M^{2}}C^{T}C$$

The calculation formula for the high-resolution image r is

(8) $$r=\frac{1}{M^{2}}C^{T}\vec{y}$$

where T represents a column vector composed of the selected M × M size window pixels, and the literature [27] indicates that M = 5, and T represents the matrix transpose symbol.

Substituting Equations (7) and (8) into Equation (6), the interpolation coefficient calculation formula is obtained as Equation (9):

(9) $$\alpha ={\left(C^{T}C\right)}^{-1}\left(C^{T}\vec{y}\right)$$

Substituting M into Equation (5) yields the missing pixel values at the edge.

Bilinear interpolation [28] is a process of sequential interpolation, where the pixel value at a desired point can be calculated using the known neighboring pixels in the image. It involves interpolating in the X-direction first and then using the interpolated X-direction result to interpolate in the Y-direction.

**Figure 6:**
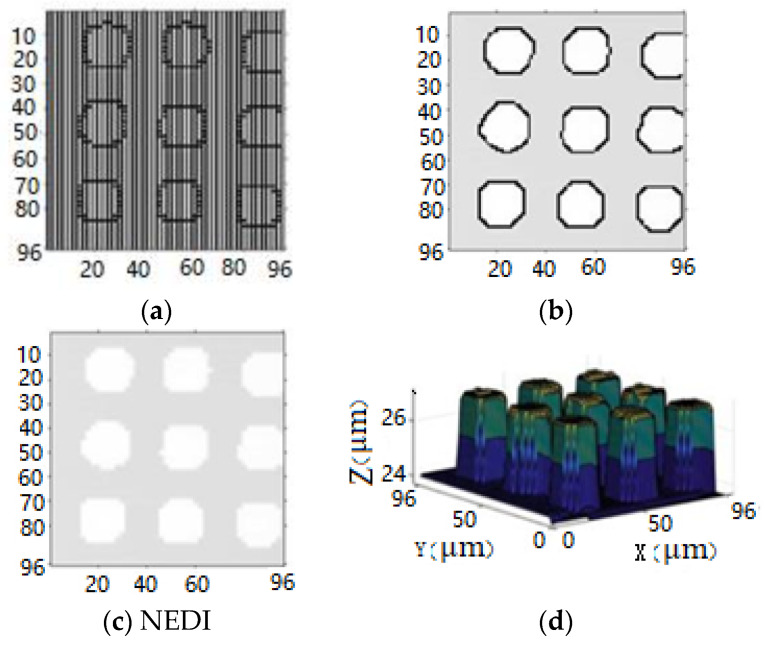
Interpolation processing and three-dimensional morphology. (**a**) Linear Interpolation in the X-axis. (**b**) Linear Interpolation in the Y-axis. (**c**) NEDI. (**d**) Enhanced three-dimensional morphology with increased resolution.

The final images and three-dimensional morphology after bilinear interpolation and NEDI processing are depicted in Figure 6.

In Figure 6, edge detection is first utilized to distinguish non-edge pixel regions, and bilinear interpolation is applied to those regions. The X-direction interpolation result is shown in Figure 6a. Subsequently, Y-direction interpolation is performed using the obtained X-interpolation result, depicted in Figure 6b. Finally, NEDI processing is applied to the image’s edge regions, resulting in Figure 6c. Converting the two-dimensional grayscale image into a three-dimensional morphology yields the resolution-enhanced three-dimensional morphology, as illustrated in Figure 6d.

## 5. An Imaging Experiment of Articular Cartilage

The superior imaging capability of SICM lies in its non-destructive three-dimensional imaging of live cells. Articular cartilage plays a crucial role in the biological activities of both animals and humans. Investigating the morphological changes on the surface of articular cartilage is significant for elucidating the occurrence and development mechanisms of various types of arthritis in the biological system [29,30,31]. Therefore, this section selects articular cartilage as the subject for relevant experiments.

### 5.1. Experimental Conditions

**Figure 7:**
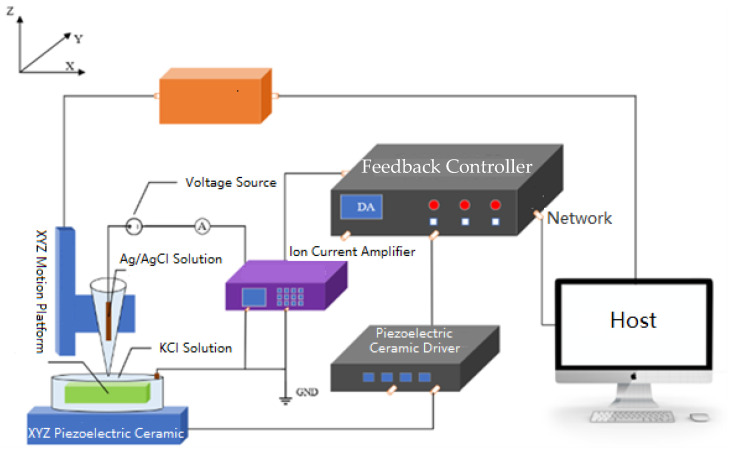
The SICM system’s configuration.

The experiment utilized a custom-built SICM scanning imaging system developed by the team, which includes an XYZ motion platform, a piezoelectric ceramic, an SICM feedback controller, an ion current amplifier, and a host computer [28]. The system configuration is illustrated in Figure 7. The sample material used was articular cartilage, and the solution employed was a KCl solution with a concentration of 0.1 M. The glass microelectrode was a single-barrel type with a diameter of 200 nm and an inner tip orifice diameter of 30 nm (NARISHIGE PC-100, pulled under a gravity of 3.646 N, and heated to 62 °C using a single-step pulling method). The electrode selected was an Ag/AgCl electrode.

### 5.2. Evaluation Metrics

This paper selects the imaging time as evaluation metric 1 and the peak signal-to-noise ratio (PSNR) of the image as evaluation metric 2. The PSNR is commonly used to indicate the quality of denoising in noisy images, and its definition is given by Equations (10) and (11):

(10) $$\mathrm{PSNR}=10\times \log_{10}\left(\frac{{\left(2^{B}-1\right)}^{2}}{\mathrm{MSE}}\right)$$

(11) $$\mathrm{MSE}=\frac{1}{2M\times 2N}\sum_{i=1}^{2M}\sum_{j=1}^{2N}||I_{0}(i,j)-I(i,j)||$$

In the equation, B’s value is 8, indicating each pixel is composed of 8 bits; MSE is the mean squared error, and the image size is 2 M × 2 N; $I_{0}$ is the real image, I is the noisy image. In this case, as obtaining the real image directly is difficult, the average of the denoised images from 50 scans is taken to be the real image, defined as follows:

(12) $$I_{0}(i,j)=\frac{1}{50}\sum_{k=1}^{50}I_{k}(i,j)$$

This paper adopts the method of calculating the PSNR of the images from 50 scans and taking the average, and the calculation formula is as follows:

(13) $$\bar{\mathrm{PSNR}}=\frac{1}{50}\sum_{i=1}^{50}{\mathrm{PSNR}}_{i}$$

This paper selects the structural similarity (SSIM) index as evaluation metric 3. The SSIM is a comparison based on three metrics of two images: their luminance, contrast, and structure. The definition of the SSIM is as follows:

(14) $$\mathrm{SSIM}(I_{2x},I_{zq})=\frac{\left(2\mu_{I_{2x}}\mu_{I_{zq}}+c_{1}\right)\left(2\sigma_{I_{2x}I_{zq}}+c_{2}\right)}{\left(\mu_{I_{2x}}^{2}+\mu_{I_{zq}}^{2}+c_{1}\right)\left(\sigma_{I_{2x}}^{2}+\sigma_{I_{zq}}^{2}+c_{2}\right)}$$

In the equation, $I_{2x}$ is a traditional 2× resolution scanning image, $I_{zq}$ is a resolution-enhanced image, $\mu_{I_{2x}}$ is the pixel mean of a traditional 2× resolution scanning image, $\mu_{I_{zq}}$ is the pixel mean of a resolution-enhanced image, $\delta_{I_{2x}}$ is the pixel variance of a traditional 2× resolution scanning image, $\delta_{I_{zq}}$ is the pixel variance of a resolution-enhanced image, and $\sigma_{I_{2x}I_{zq}}$ is the covariance of pixels between a traditional 2× resolution scanning image and a resolution-enhanced image. $c_{i}={\left(k_{i}L\right)}^{2}$ (i=1,2) is a constant, and $L=2^{B}-1$, $k_{1}=0.01$, and $k_{2}=0.03$ is the default value.

This paper adopts the method of calculating the SSIM of the images from 50 scans and taking the average, and the calculation formula is as follows:

(15) $$\bar{\mathrm{SSIM}}=\frac{1}{50}\sum_{i=1}^{50}{\mathrm{SSIM}}_{i}$$

### 5.3. Experiment and Analysis

#### 5.3.1. The SICM High-Speed Scanning Reconstruction Imaging of Joint Cartilage at Two Resolutions

To compare the imaging time and quality of different methods, we first sampled a joint cartilage specimen using the SICM scanning imaging system described in Section 4. In the experiment, the probe movement speed was set to 480 nm/ms in high-speed scanning mode.

**Figure 8:**
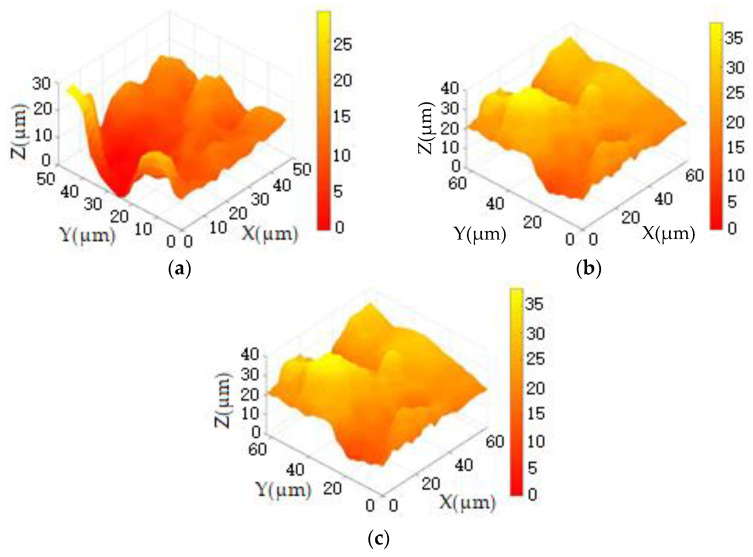
The imaging of the joint cartilage sample (Region 1#, Region 2#, Region 2#). (**a**) 48 × 48 pixels. (**b**) 64 × 64 pixels. (**c**) 100 × 100 pixels.

1 Utilizing the traditional full sampling-scanning method with a step distance of 1 μm, we imaged selected areas on the surface of the joint cartilage specimen, with the results shown in Figure 8. Through piezoelectric ceramic measurement, the maximum height difference in the scanned area of the specimen was found to be 40 μm. Specifically, Figure 8a presents the imaging of region 1# of the cartilage specimen, where the maximum height difference is 30 μm, the imaging area is 48 × 48 μm^2^, and the number of imaging pixels is 2304. Figure 8b illustrates the imaging of region 2# of comprehensive experiment 53 of the cartilage specimen, with a maximum height difference of 40 μm, an imaging area of 64 × 64 μm^2^, and the number of imaging pixels being 4096. Figure 8c displays the imaging of another region of cartilage specimen 2#, where the maximum height difference is 40 μm, the imaging area is 100 × 100 μm^2^, and the number of imaging pixels is 10,000. Through the above imaging results, we can evaluate the imaging effects of the traditional full-sampling scanning method at different resolutions, providing benchmark data for subsequent comparisons. This will help us to better understand the performance differences of different imaging methods.

**Figure 9:**
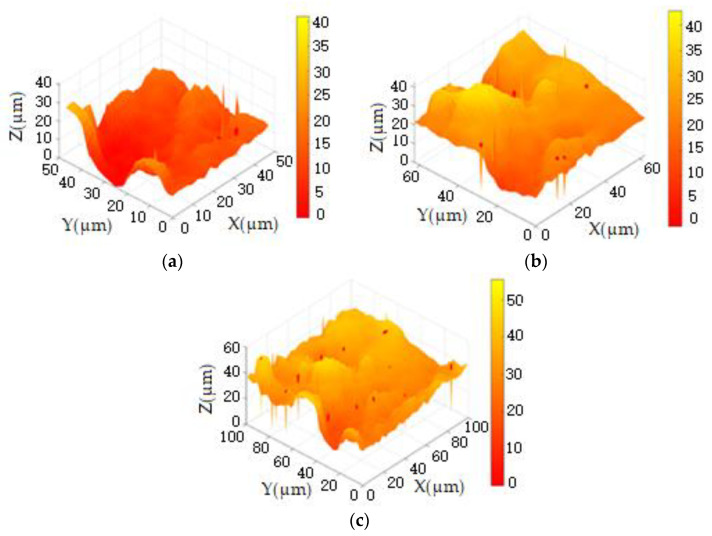
The scanning imaging of articular cartilage at (**a**) 48 × 48 pixels, (**b**) 64 × 64 pixels, and (**c**) 100 × 100 pixels at 1× resolution.

2 The imaging results of the selected areas on the surface of the joint cartilage specimen reconstructed using the high-speed undersampling scanning method with a step distance of 1 μm are shown in Figure 9.

From Figure 9, it can be observed that under the high-speed undersampling scanning reconstruction mode, the three-dimensional morphology of the joint cartilage with sizes of 48 × 48 pixels, 64 × 64 pixels, and 100 × 100 pixels all exhibit significant noise, with the number of noise points increasing with the pixel count. According to the noise threshold set by the detection threshold of the ion current’s decrease exceeding a predefined threshold ($I_{d}<0.99I_{0}$), there are 13 noise points in image (a), 21 noise points in image (b), and 43 noise points in image (c). The real appearance of the image at the noise point is covered up, and there are a certain number of burrs on the surface of the appearance, which makes it difficult to meet the requirements of standard analysis.

**Figure 10:**
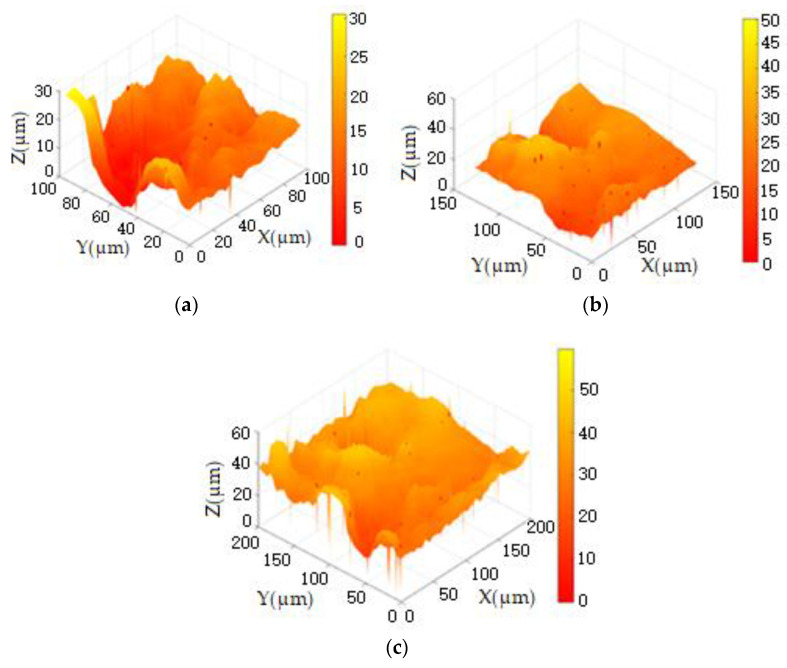
The scanning imaging of articular cartilage at (**a**) 96 × 96 pixels, (**b**) 128 × 128 pixels, and (**c**) 200 × 200 pixels at 2× resolution.

3 To capture more details of the surface morphology, the probe step distance was set to 0.5 μm. Employing the 2× resolution as shown in Figure 9 (96 × 96 pixels, 128 × 128 pixels, 200 × 200 pixels) with the high-speed undersampling scanning mode, the selected regions of the joint cartilage sample’s surface were reconstructed for imaging, as illustrated in Figure 10.

From Figure 10, it is evident that imaging with 2× resolution compared to 1× resolution in Figure 9 resulted in more noise points. In image (a), there are 32 noise points, 57 in image (b), and 96 in image (c). Although the 2× resolution provides more details of surface morphology, it also introduces more noise points, consequently masking more genuine morphological features. Experimental evidence indicates that the imaging time with 2× resolution significantly exceeds that of 1× resolution.

By comparing Figure 9 and Figure 10, it is evident that the overall morphology of the image remains unchanged before and after increasing the scanning resolution. While 2× resolution provides clearer morphological details, it also reduces the imaging time resolution. Therefore, considering both factors, it is advisable to opt for 1× resolution scanning for reconstruction imaging and employ algorithms to enhance image resolution.

#### 5.3.2. Resolution Enhancement of Scanned Images at 1× Resolution

**Figure 11:**
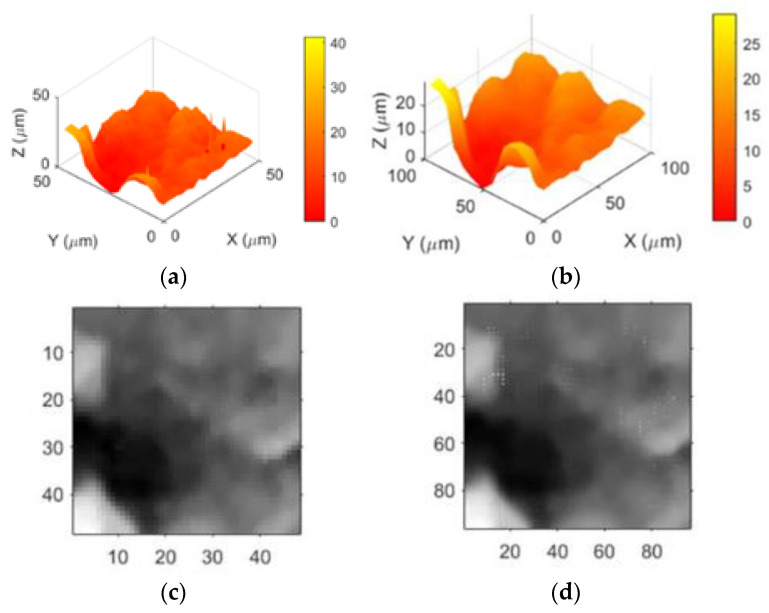
The interpolation and 3D morphology of a 48 × 48 pixel image. (**a**) Reconstructed morphology (1× resolution). (**b**) Enhanced three-dimensional morphology (2× resolution). (**c**) Reconstructed morphology of a local grayscale image. (**d**) Image after NEDI algorithm processing.

**Figure 12:**
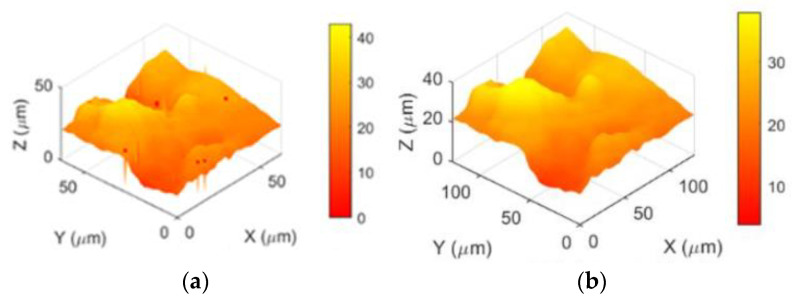

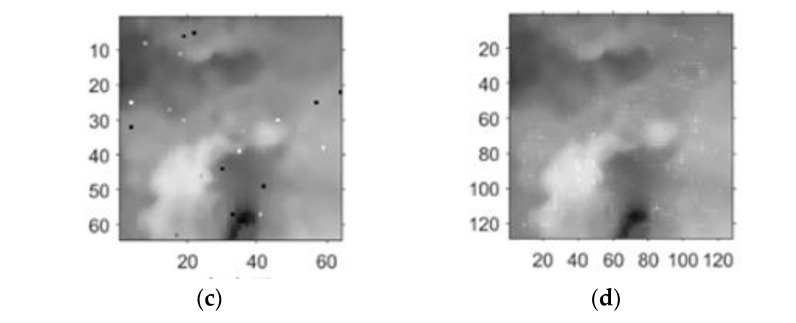
The interpolation and 3D morphology of a 64 × 64 pixel image. (**a**) Reconstructed morphology (1× resolution). (**b**) Enhanced three-dimensional morphology (2× resolution). (**c**) Reconstructed morphology of a local grayscale image. (**d**) Image after NEDI algorithm processing.

**Figure 13:**
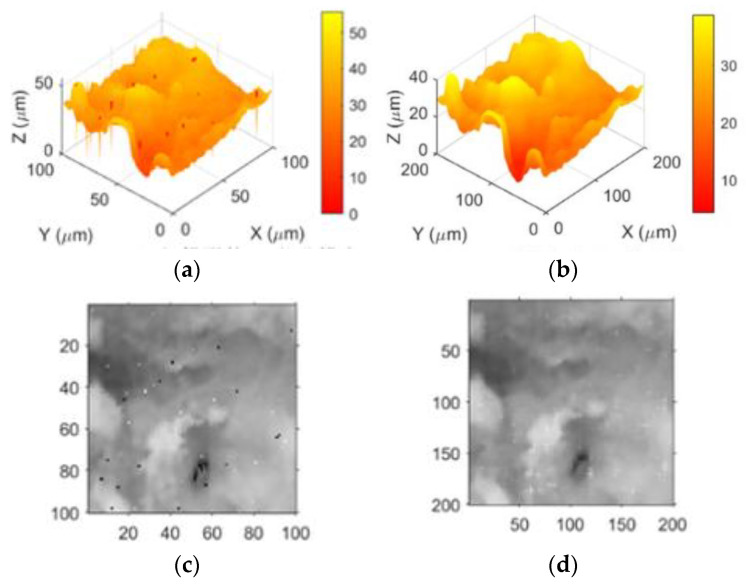
The interpolation and 3D morphology of a 100 × 100 pixel image. (**a**) Reconstructed morphology (1× resolution). (**b**) Enhanced three-dimensional morphology (2× resolution). (**c**) Reconstructed morphology local grayscale image. (**d**) Image after NEDI algorithm processing.

This experiment utilized joint cartilage samples with a resolution of 1×. To ensure the reliability and stability of the experimental results and reduce the variability caused by accidental errors, fluctuations in experimental conditions, or other uncertainties in individual experimental outcomes, scanning and imaging were performed 50 times for three different pixel-sized regions. The high-speed scanning was conducted at 480 nm/ms. A total of 150 scan images were obtained, comprising 50 sets, each with initial image sizes of 48 × 48, 64 × 64, and 100 × 100 pixels, and a resolution of 1 μm. Subsequently, MATLAB R2018b software was employed to apply resolution enhancement algorithms to the scan images, resulting in processed images for each of the three sizes. One set of these processed images is presented in Figure 11, Figure 12 and Figure 13.

First, the three-dimensional reconstructed morphology of joint cartilage at 1× resolution is obtained, as shown in Figure 10, Figure 11 and Figure 12a. It can be observed from the images that there is considerable noise in the scanned morphology, and the noise at the sample morphology leads to distortion, affecting subsequent analyses. Therefore, the image is subjected to grayscale processing, resulting in the grayscale image shown in (c), followed by denoising using median filtering. To separately apply NEDI processing to the edges, edge detection is performed on the denoised image using the Canny operator, and the detected edges are expanded by a factor of two to serve as the interpolation image. The interpolation process is described as follows: first, using the edges detected by the Canny operator as a condition for differentiation, bilinear interpolation is applied to non-edge pixels. Initially, interpolation is done in the X-direction, and the result is then used for interpolation in the Y-direction. Subsequently, NEDI processing is applied to the edges, completing edge pixel filling and the entire interpolation process, resulting in the resolution-enhanced two-dimensional scanned image shown in (d). Furthermore, to eliminate the influence of singular values causing a small number of noise points in the NEDI calculation process, the image undergoes median filtering once again. Finally, the image is transformed into three dimensions, yielding the reconstructed image with enhanced resolution in three dimensions, as shown in (b). In image (b), noise points are removed, and the resolution is significantly improved, making local microscopic details clearer. If the above process is iterated N times, it can achieve a 2N-fold enhancement in image resolution, thereby providing an alternative means to increase the imaging speed of SICM.

Analyzing the processing results leads to the following preliminary conclusions: (1) while maintaining a high scanning speed, the resolution of the scanned images is enhanced, equivalent to improving the imaging efficiency; (2) scanned morphology noise is effectively filtered out, and the true morphology is restored at the original noisy locations; (3) the three-dimensional morphology of the resolution-enhanced images is, overall, similar to the scanned morphology, indicating that the proposed algorithm does not sacrifice image quality.

#### 5.3.3. Indicator Analysis

To validate the above conclusions, this paper first compares the imaging times of the three imaging types, as shown in Table 2.

From the statistical results in Table 2, the imaging times of the resolution-enhanced images obtained under 1× resolution + resolution enhancement are 26 min, 40 min, and 93 min, respectively. The imaging time is almost the same as that of traditional 1× scanning resolution and is reduced by 67.08%, 72.02%, and 74.66% compared to that of traditional 2× scanning resolution, reducing by at least 60%. Thus, the SICM imaging efficiency is significantly improved.

We calculated the average PSNR and SSIM of the scanned images 50 times, and the calculation results for the $\bar{\mathrm{PSNR}}$ are shown in Table 3.

The calculation results from Table 3 indicate that for articular cartilage images of sizes 96 × 96 pixels, 128 × 128 pixels, and 200 × 200 pixels at 2× resolution, the $\bar{\mathrm{PSNR}}$ values are 34.8201, 35.7524, and 42.9047, respectively. At 1× resolution with the algorithm proposed in this paper, the $\bar{\mathrm{PSNR}}$ values are 43.3997, 43.2420, and 52.9047, representing an improvement of 7 dB compared to 2× resolution. This objectively demonstrates that the SICM scanned morphology, after median filtering, exhibits reduced image noise and increased effective information, achieving good denoising effects.

Furthermore, the average SSIM between the scanned morphology and the resolution-enhanced morphology from 50 scans was calculated. The SSIM index can be used to evaluate the similarity between two images, with a range of −1 to 1, where 1 indicates that the two images are identical. The $\bar{\mathrm{SSIM}}$ calculation results are shown in Table 4.

From the calculation results in Table 4, it can be observed that the structural similarity index $\bar{\mathrm{SSIM}}$ values of the reconstructed images of the joint cartilage with 50 iterations at 2× resolution, 1× resolution, and 1× resolution with resolution enhancement are 0.9879, 0.9784, and 0.9801, respectively. This indicates that the similarity between the images under the three imaging conditions is greater than 97%. Therefore, this demonstrates that the proposed method in this study can enhance the scanning image resolution without sacrificing image quality.

## 6. Conclusions

This paper addresses the issue of a reduced signal-to-noise ratio with an increased scanning speed in the jump mode of SICM, as well as the problem of a low imaging time resolution at low speeds. It proposes an algorithm for the denoising and resolution enhancement of images under high-speed scanning mode, resolving the contradictory relationship between the imaging time resolution and the spatial resolution of SICM. The experimental SICM imaging of articular cartilage demonstrates that, after processing with the algorithm proposed in this paper, the imaging time resolution is improved by 60% compared to traditional 2× resolution scanning imaging, and the peak signal-to-noise ratio of the scanned images is increased by 7 dB, with image similarity being as high as 97%. Therefore, the proposed algorithm effectively enhances the imaging efficiency and quality of SICM without sacrificing image quality, holding significant application value in the study of biological cell pathology and unlocking the mysteries of life.

## Figures and Tables

**Table 1:** The image peak signal-to-noise ratio under each algorithm.

Algorithm	Reconstructed Morphology	Gaussian Filtering	Mean Filtering	Median Filtering	Wavelet Filtering
PSNR/dB	29.3176	30.5351	33.4893	37.1769	31.8092

**Table 2:** Imaging times for three imaging types.

Imaging Modality	Pixel	Imaging Time
1× Resolution	48 × 48 pixels	21 min
	64 × 64 pixels	36 min
	100 × 100 pixels	91 min
2× Resolution	96 × 96 pixels	79 min
	128 × 128 pixels	143 min
	200 × 200 pixels	367 min
1× Resolution + Resolution enhancement	96 × 96 pixels	26 min
	128 × 128 pixels	40 min
	200 × 200 pixels	93 min

**Table 3:** The results of the PSNR mean being calculated 50 times.

Imaging Modality	Pixel	$\bar{\mathrm{PSNR}}/\mathrm{dB}$ (Mean of 50 Times)
2× Resolution	96 × 96 pixels	34.8201
	128 × 128 pixels	35.7524
	200 × 200 pixels	42.9047
1× Resolution + Resolution enhancement	96 × 96 pixels	43.3997
	128 × 128 pixels	43.2420
	200 × 200 pixels	52.9047

**Table 4:** The SSIM mean of 50 times.

Imaging Modality	Pixel	$\bar{\mathrm{SSIM}}$ (Mean of 50 Times)
2× Resolution	96 × 96 pixels	0.9879
1× Resolution	48 × 48 pixels	0.9784
1× Resolution + Resolution enhancement	96 × 96 pixels	0.9801

## Data Availability

Data are contained within the article.
